# An Innovative Youth‐Friendly Electronic Questionnaire to Identify Mental, Sexual and Reproductive Health Risks: A Validation Study of the Total Teen Assessment

**DOI:** 10.1111/hex.70313

**Published:** 2025-08-07

**Authors:** Whitney R. Garney, Kelly L. Wilson, Meg Patterson, Caitlin B. Holden, Sara Flores, Sonya Panjwani, Kristen Garcia, Christi Esquivel, Ashley Khanhkham, Kobi V. Ajayi

**Affiliations:** ^1^ School of Public Health Texas A&M University College Station Texas USA; ^2^ School of Nursing Texas A&M University College Station Texas USA

**Keywords:** adolescent health, mental health, psychometrics, sexual health, substance use

## Abstract

**Background:**

The American Medical Association and the American Academy of Pediatrics recommend screenings during adolescents' annual preventive health appointments. However, the literature reports only 50% completion at their last appointment, with less than half of providers discussing recommended health topics. Barriers such as time constraints and discomfort contribute to low completion rates. The Total Teen (TT) Assessment is a novel approach utilising an online questionnaire to assess adolescent health needs related to sexual and reproductive health, mental health and substance use behaviours.

**Methods:**

A three‐phase psychometric development and validation study was completed to investigate the TT Assessment's validity and reliability. Phase 1 focused on scale development, Phase 2 validated content with patients and healthcare providers and Phase 3 utilised psychometric testing to ensure the scale adequately assessed its intended domains.

**Results:**

Parallel analysis conducted on the correlation matrix extracted three total factors: Factor 1: sexual health, Factor 2: mental health and Factor 3: substance use. The TT Assessment is a statistically and clinically valid instrument used to easily evaluate adolescent needs related to sexual, reproductive and mental health. Quantitative psychometric analyses revealed its soundness, while qualitative investigations revealed its clinical relevance and diagnostic accuracy.

**Conclusions:**

The TT Assessment is an innovative instrument that can be easily adapted to assess the needs of adolescent patients, as their health needs often go unnoticed until they require severe clinical intervention. The TT Assessment helps providers identify sexual, reproductive and mental needs early so they can help reduce the likelihood of major issues.

## 1 Introduction

Adolescents experience intense social, emotional and physical changes as they transition from childhood to adulthood [1]. As adolescents' brains rapidly develop, they must navigate biological changes, emotional learning and new social dynamics. While most adolescents successfully endure, some are unable to manage sudden changes, regulate their emotions or cope with daily stressors, which can lead to maladaptive behaviours such as risky sexual practices, violence and/or mental health issues. For example, risky sexual practices among adolescents (aged 15–24 years) cause approximately half of all sexually transmitted infections (STIs) in the United States [2]. In addition, the prevalence of intentional/unintentional injuries and mental health disorders increases during adolescence [2, 3].

While these are complex public health issues, developmentally appropriate, robust screening in primary care settings could mitigate these challenges by providing a forum for dialogue, medically accurate information and supportive services for adolescents. The American Medical Association and the American Academy of Pediatrics recommend that adolescents be screened for sexual health, mental health and substance use during annual preventive health appointments [4, 5]. Unfortunately, traditional clinical practices do not always provide comprehensive, developmentally appropriate screening for adolescents. A recent national survey revealed that only ~50% of adolescents completed a health questionnaire at their last appointment, and less than half reported discussing recommended health topics [6].

Social and ecological barriers to adolescent preventative health screening have been well‐documented in previous literature. Screening practices are influenced by barriers at the organisation, provider and patient levels [7]. Competing demands create logistical barriers like a lack of visit time or appropriate referral partners [8]. Current validated screening instruments require providers to possess interpersonal skills to facilitate screening, interpret results and intervene appropriately [9]. Screenings resulting in honest feedback are most successful in confidential environments; however, providers and adolescents report concern regarding confidentiality and honesty. Evidence suggests adolescents are less willing to discuss health concerns when confidentiality is not ensured [10]. Confidentiality and privacy are critical to screening and treating adolescents for potentially sensitive health concerns, especially in mental health and sexual reproductive health screenings [11]. However, organisational and/or state policies often limit or prohibit opportunities for confidential visit time for adolescents.

Furthermore, screening modality and timing matter. Adolescent patients would rather complete screenings privately in a clinic than before an appointment or in real time with a provider [12]. They also prefer electronic assessment; when used, they report higher levels of comfort and honesty than paper or provider‐administered questionnaires [12]. Research indicates that electronic health assessment improves adolescents' perceptions of clinician communication and partnership and produces more accurate data [7, 13].

Efficient screening processes, such as intentional clinical workflow integration, confidential time and electronic screening tools, can better facilitate youth‐friendly screening in healthcare settings [8].

### 1.1 The TT Programme

The Total Teen (TT) programme was developed to overcome barriers to screening, as well as to improve adolescent access to and experience with healthcare [14, 15, 16, 17]. The programme can be implemented for wellness and sick visits in primary care settings. It includes a tailored clinical workflow, a youth‐friendly screening for adolescents (ages 12 and older), immediate sexual and/or behavioural health services, and referrals or follow‐up services as needed. The programme is based on a youth‐friendly approach, which the World Health Organization identifies as accessible, acceptable, appropriate and effective health services designed to meet the diverse needs of adolescents and young adults [18]. This paper reports a three‐phase psychometric study to validate the TT programme's youth‐friendly, developmentally appropriate health screener, the TT Assessment.

## 2 Materials and Methods

### 2.1 Study Design

To determine the validity of the TT Assessment, researchers conducted a three‐phase psychometric study. Phase 1 focused on scale development, Phase 2 validated content with patients and healthcare providers and Phase 3 utilised psychometric testing to ensure the scale adequately assessed its intended domains. Across all stages, researchers collaborated iteratively with stakeholders, involving health professionals serving adolescents in diverse healthcare settings and adolescents who served on a project advisory committee. The receipt and analysis of the data used in this study were approved under the Texas A&M University Institutional Review Board.

### 2.2 TT Assessment

The TT Assessment was a self‐completed, electronic questionnaire designed to assess adolescent health needs across multiple domains, including three sections dedicated to sexual and reproductive health, mental health and substance use behaviours, respectively. The questions in each section are derived from evidence‐based tools and research (i.e., Patient Health Questionnaire‐9 [PHQ‐9], Generalized Anxiety Disorder‐2 and Screening to Brief Intervention Tool [S2Bi]) [19, 20, 21]. The assessment was administered in the clinic electronically via tablet to ensure confidentiality and to support adolescents' communication preferences. Skip logic was used to display relevant questions based on patients' responses. Scores for each section were automatically generated and displayed on the tablet in a youth‐friendly format immediately after assessment completion. Adolescent patients were classified into no‐to‐low or moderate‐to‐high need categories based on their scores, which guide clinical intervention. The assessment questionnaire comprised items measuring sexual and reproductive health risk, mental health risk and substance use risk.

### 2.3 Phase 1: Scale Development

Following iterative consultation with project stakeholders, researchers developed the TT Assessment by reviewing recent research on sexual risk, mental health questionnaires and substance use assessments. This process narrowed the key content domains into three overarching groupings: sexual and reproductive health, mental health and substance use.

#### 2.3.1 Sexual and Reproductive Health Questions

Eleven items were adopted from three existing instruments to measure sexual and reproductive health risks. The data were summed and scored into low (0–4) or mod/high risk (5–13) categories. First, one item was adopted from the Youth Risk Behavior Surveillance Survey (YRBSS) created by the Centers for Disease Control and Prevention (CDC) to monitor the health behaviours of youth and young adults that contribute to death, disability and social problems [22]. The 2019 YRBSS included 99 items covering 121 health‐related behaviours, including sexual health, mental health and many others [22]. Next, six items were adopted from the National Survey of Adolescents and Young Adults: Sexual Health Knowledge, Attitudes and Experiences [23]. The Henry J. Kaiser Family Foundation (KFF) created the National Survey of Adolescents and Young Adults: Sexual Health Knowledge, Attitudes and Experiences to move beyond the ‘standard questions about sexual behavior’ and directly assess the thoughts of young people by asking them questions about their knowledge and attitudes towards sex and sexual health [23]. Lastly, four items were adopted from the Teen Sexual Health Survey created to evaluate a comprehensive adolescent sexual health education programme called Chicago Healthy Adolescents and Teens (CHAT) [24]. The National Opinion Research Center (NORC) at the University of Chicago developed the instrument, and the survey comprises 64 items that focus on knowledge and behaviour related to healthy sexual practices and pregnancy and STI prevention [24].

#### 2.3.2 Mental Health Questions

Mental health was measured using items from two existing instruments. First, to measure mental health risk, 10 items were adopted from the existing PHQ‐9 [20]. The PHQ‐9 was derived from an assessment tool utilised by medical professionals called the Primary Care Evaluation of Mental Disorders (PRIME‐MD) that addresses five of the most common types of mental disorders presenting in medical populations: depressive, anxiety, somatoform, alcohol and eating disorders [20]. The data were summed and scored into low (1–4), mild (5–9), moderate (10–14), moderate/severe (15–19) and severe categories (20–27). Three items were adopted from the existing Generalized Anxiety Disorder two‐question scale (GAD‐2) [21] to measure anxiety disorder. The GAD scales also originated from the PRIME‐MD [21]. The data were summed and scored as low (0–2) or high risk (3+).

#### 2.3.3 Substance Use Questions

Nine items were adopted from the S2BI [19] to measure substance use risk. S2BI is used to assess the frequency of alcohol and substance use among adolescents and was developed by Boston Children's Hospital and validated in 2014 [19]. Data were summed and scored as low (0: never or once/twice), moderate (2: monthly) or severe (3: weekly) [19].

### 2.4 Phase 2: Content Validation

To determine the assessment's content validation, we asked youth and healthcare professionals to review and critique the questions and overall instrument. We conducted two focus groups (*n* = 8) via Zoom in September and October 2021 to gather youth feedback. Participants were recruited from the youth advisory group. The sample was predominantly female (97.5%), with one informant identifying as non‐binary and no male representation. Informants ranged from 12 to 17 years old and represented five states (i.e., Texas, Colorado, Connecticut, Florida and Louisiana). A qualitative researcher facilitated both focus groups, and two other research team members observed and documented field notes. The facilitator presented the TT Assessment in sections by topic, prompting informants to discuss content coverage related to their lived experiences and to comment on its appropriateness for use with adolescent patients regarding format, verbiage, clarity and length. Informants were also prompted to propose ways to revise items they felt required rephrasing or reformatting. Throughout the process, the moderator paraphrased the informant's statements to provide opportunities for clarification. Each focus group lasted between 30 and 40 min.

Assessment feedback was also gathered from healthcare professionals at the five healthcare organisations who were implementing partners for this study. Healthcare organisations were located in four states (Connecticut, Hawaii, Illinois and Texas). They represented a variety of organisation types, including a Federally Qualified Health Center (FQHC), school‐based health clinics, an adolescent health clinic and a women's specialty clinic. We collected feedback through one‐on‐one calls with representatives from each organisation. Organisational professionals providing feedback differed by organisation and included primary care and specialty healthcare providers, mental health providers, other clinic personnel and administrative personnel. Before the calls, organisations received the full survey and were instructed to review and provide feedback on necessary changes. Healthcare organisations were specifically asked whether their youth patients would comprehend the questions and answer choices, if terminology would be considered outdated or offensive, if questions should be omitted entirely, and general feedback on the flow and expected acceptability for clinicians and patients. Changes were made to the survey before it was administered to the youth. Throughout and following the implementation process, we gathered additional feedback on problematic questions and recommended changes.

### 2.5 Phase 3: Psychometric Analysis

#### 2.5.1 Settings and Participants

Five healthcare organisations participated in a 6–10 week implementation period in Spring 2022. Organisations represented settings where youth accessed healthcare services, including an adolescent health clinic, an FQHC, a women's specialty clinic and two school‐based health clinics. Assessment participants were adolescent patients (*n* = 481), aged 12–25, who attended the clinics for non‐emergency‐related care. All clinics treated the assessment as a diagnostic tool to supplement normal practice; therefore, all patients were assessed privately. They were informed that participation was voluntary and that they could refuse to take the survey.

#### 2.5.2 Quantitative Data Collection and Analysis

The TT Assessment was administered electronically via iPad and Qualtrics software. Each clinic determined the most appropriate time to incorporate the assessment into its clinical workflow. Most clinics asked patients to complete the assessment while waiting for the provider or during their vital check. Results for the three domains of risk categories were automatically calculated at the end of the assessment so the patient and provider could review and document the results. Providers could also access item responses if they wanted specific assessment information. Data was sent to the healthcare entity (via Qualtrics email) so clinic staff could chart scores into their electronic health records. Each organisation downloaded and de‐identified the TT Assessment responses at the end of the implementation period and sent them to the research team for analysis. To test the psychometric properties of the TT Assessment, we first conducted an exploratory factor analysis (EFA) to determine the structure of underlying factors within our set of items [25]. Then, we conducted a confirmatory factor analysis (CFA) to test the fit of the factors discovered in the EFA.

##### 2.5.2.1 EFA

EFA is an analytical technique that reveals underlying factors among a set of observed variables [25]. Because our data was ordinally scaled, we used Spearman correlations and the Weighted Least Squares estimation method to explore latent factor structures in our data. Factor structures were evaluated using parallel analysis, which compares eigenvalues obtained from the empirical correlation matrix to the eigenvalues obtained from simulated datasets of equal size [26]. Eigenvalues were initially set to one, and we ran a scree plot [27] to determine cut points that would reveal latent structures within the observed data. The initial scree plot suggested eigenvalues of 1.5 or higher were most noteworthy, and as a result, we re‐ran the EFA with eigenvalues set to 2. Next, we reviewed the communalities matrix to identify variables that were not contributing to the resulting set of factors. Based on the communalities matrix, we reduced the observed variables to focus on the most meaningful items. A third and final EFA was conducted, including observed values that were significant in the communalities matrix using eigenvalues set at 2 for parallel analysis. Finally, we computed Cronbach's *α* values on each factor to evaluate internal consistency. EFAs were conducted in SPSS version 27 [28].

##### 2.5.2.2 CFA

CFA assesses the fit of the underlying factors identified in an EFA. We assessed goodness of fit using the Comparative Fit Index (CFI), the Root Mean Square Error of Approximation (RMSEA) and the Standardised Root Mean Square Residual (SRMR). Goodness of fit is revealed when CFI values are greater than or equal to 0.95, RMSEA values are less than or equal to 0.07 and SRMR values are less than 0.08. Using the lavaan package [29] in R statistical software [30], we conducted a CFA on the three factors identified in the EFA.

#### 2.5.3 Qualitative Data Collection and Analysis

Since the TT Assessment was meant to help providers make diagnostic decisions, the results needed to be useful to clinicians. To evaluate the diagnostic integrity of items, clinicians were consulted to determine if TT Assessment scores paralleled their professional diagnosis.

Five interviews were conducted with the clinicians who administered the TT Assessment. Both sexual and reproductive health and mental health professionals participated (*n* = 5) in the interviews. Providers were asked, ‘*In what ways does this program fit the needs of your patients?’* Probes included, ‘*Tell me about your experience with the TT Assessment.’* and ‘*Did you feel like the TT assessment scores were comparable to your clinical opinion?’* Data was audio recorded and documented in field notes and then analysed using a thematic analysis. To analyse the field notes, the lead evaluator, who did not serve as an interviewer, reviewed the notes, identified codes on selected questions for each individual interview and then combined the findings across interviews to develop themes [31]. Emergent codes were identified using an inductive approach rather than categorising themes using a predefined guide [31].

## 3 Results

### 3.1 Quantitative Results

As seen in Table 1, among the 481 patients, over half (56.1%) were females, with ages ranging from 12 to 25 years old and a mean age of 16.5. The majority identified as having straight sexual orientation (77.8%) and as Asians (22.9%).

**Table 1:** Socio‐demographic characteristics of youths.

Variables	*N* (%)
Age	476 (98.9)
Range, mean, standard deviation	12–25, 16.5, 2.4
Sex	481 (100)
Male	177 (36.7)
Female	270 (56.1)
Others	1 (0.2)
Missing	33 (6.7)
Sexual orientation	455 (94.5)
Straight	354 (77.8)
LGBTQIA+	101 (22.1)
Missing/Prefer not to say	26 (5.7)
Race	481 (100)
White or Caucasian	57 (11.6)
Black or African American	60 (12.5)
Hispanic or Latino	86 (17.9)
Asians	110 (22.9)
American Indian or Alaska Native	7 (1.5)
Native Hawaiian or Other Pacific Islander	92 (19.1)
Multiracial or Biracial	28 (5.8)
Others	41 (8.5)

Descriptive statistics for each item are presented in Table 2. Specifically, the mean value for Items 1–16, which assessed sexual and mental health, ranged from 0.21 to 1.20, suggesting variability in endorsing these items. The mean values for substance use items (17–22), which were scored 0–3, ranged from 0.05 (ever overdosed) to 0.68 (ever used marijuana), indicating most in the sample had not used substances. Skewness for the sexual and mental health items demonstrates positive and negative distributions, ranging from −0.68 to 2.55. Skewness for substance use ranged from 1.17 to 7.80, which also suggests most respondents had lower substance use, pulling the sample distribution mean to the right. Lastly, kurtosis values for the sexual and mental health items indicated varied distributions, ranging from −1.98 to 4.78. Substance use variables also had variability in their kurtosis distributions, with alcohol and marijuana use registering flatter distributions (0.18 and 0.25, respectively), whereas tobacco use, prescription drug use and ever overdosing registered leptokurtic kurtosis distributions (ranging from 14.56 to 24.16).

**Table 2:** Item descriptive statistics.

Item	*M* ^a^	SD^b^	Skewness	Kurtosis
1. Ever had a sexual experience	0.39	0.52	0.79	−0.68
2. Used contraceptives	0.54	0.50	−0.17	−1.98
3. Used condoms	0.21	0.59	2.55	4.78
4. Learn more about sexual health	0.23	0.42	1.29	−0.34
5. Little interest or pleasure in doing things	0.85	0.93	0.86	−0.23
6. Feeling down, depressed, irritable or hopeless	0.74	0.90	0.95	−0.12
7. Trouble falling asleep, staying asleep or sleeping too much	1.18	1.08	0.46	−1.06
8. Feeling tired or having little energy	1.20	1.00	0.40	−0.91
9. Poor appetite, weight loss or overeating	0.82	0.97	0.94	−0.24
10. Feeling bad about yourself…	0.78	0.99	1.02	−0.15
11. Trouble concentrating…	1.04	1.07	0.61	−0.93
12. Moving or speaking slowly…	0.55	0.91	1.52	1.15
13. Thoughts that you would be better off dead	0.96	1.02	0.72	−0.66
14. Feeling nervous, anxious or on edge	0.84	1.04	0.95	−0.42
15. Not being able to stop worrying	0.22	0.41	1.38	−0.11
16. Interested in speaking to a mental health provider	0.21	0.41	1.41	−0.02
17. Used tobacco in the last year	0.16	0.61	4.10	16.03
18. Used alcohol in the last year	0.67	0.94	1.17	0.18
19. Used marijuana in the last year	0.68	1.09	1.35	0.25
20. Used vaping in the last year	0.41	0.88	7.80	3.44
21. Used prescription drugs that were not prescribed for you in the last year	0.12	0.47	4.77	24.16
22. Ever overdose	0.05	0.22	4.06	14.57

^a^ Mean.

^b^ Standard deviation.

Parallel analysis conducted on the correlation matrix (Table 3) extracted three total factors, accounting for 69.39% of the total variance (see Table 4 for EFA results). Items 1–4 assessed sexual health, demonstrating weak to moderate intercorrelations (*r* = 0.3–0.32). In contrast, Items 6–14, which measured mental health, displayed a moderate to strong positive correlation (*r*= 0.41–0.70), suggesting high correlations in response to mental health items. The substance use items (17–22) exhibited a weak to moderate intercorrelation (0.03–0.59), suggesting there was some overlap between using various substances, but not all (i.e., using alcohol was loosely correlated with using prescription drugs).

**Table 3:** Item correlation matrix.

	1	2	3	4	5	6	7	8	9	10	11	12	13	14	15	16	17	18	19	20	21	22
1																						
2	0.09																					
3	0.03	0.18																				
4	−0.14	0.32	0.31																			
5	−0.01	−0.01	0.05	−0.01																		
6	0.08	0.06	0.08	0.04	0.54																	
7	0.08	0.02	0.14	0.06	0.41	0.56																
8	0.00	0.00	0.08	0.01	0.49	0.61	0.65															
9	0.18	0.11	0.13	0.07	0.45	0.51	0.51	0.49														
10	0.05	0.09	0.09	0.08	0.46	0.70	0.50	0.53	0.51													
11	0.01	−0.00	0.10	0.01	0.51	0.58	0.52	0.57	0.50	0.55												
12	0.02	0.04	0.14	0.11	0.49	0.46	0.41	0.42	0.43	0.48	0.62											
13	0.07	0.04	0.12	0.06	0.51	0.67	0.53	0.52	0.51	0.65	0.61	0.58										
14	0.13	0.15	0.11	0.10	0.37	0.63	0.46	0.47	0.42	0.56	0.44	0.37	0.52									
15	0.14	−0.02	0.01	0.03	0.17	0.22	0.15	0.20	0.21	0.15	0.18	0.23	0.22	0.25								
16	0.15	0.17	0.23	0.23	0.21	0.32	0.24	0.18	0.32	0.28	0.26	0.33	0.38	0.28	0.07							
17	0.24	−0.08	−0.05	−0.17	0.11	0.19	0.02	0.12	0.23	0.18	0.02	0.08	0.16	0.06	0.05	0.08						
18	0.46	−0.11	−0.06	−0.12	−0.05	0.08	0.04	0.01	0.18	−0.04	−0.01	−0.02	0.09	0.01	0.01	0.03	0.41					
19	0.39	−0.09	−0.00	0.10	−0.02	0.17	0.09	0.11	0.29	0.06	0.04	0.03	0.14	0.03	0.06	0.13	0.48	0.51				
20	0.33	0.02	−0.01	0.05	−0.02	0.08	0.08	0.04	0.31	0.04	0.04	0.11	0.17	0.05	0.05	0.01	0.48	0.43	0.59			
21	0.08	0.07	0.15	0.16	0.03	0.04	0.08	0.03	0.15	0.06	0.03	0.21	0.08	0.09	0.14	0.19	0.21	0.03	0.15	0.21		
22	0.101	0.01	0.05	0.07	0.14	0.19	0.07	0.17	0.06	0.13	0.19	0.26	0.21	0.22	0.23	0.17	0.10	0.06	0.16	0.10	0.15	

*Note:* The items in this table align with the numerical items listed in Table 1.

**Table 4:** Rotated component matrix revealing three latent factors.

Item	Factor 1: Sexual health	Factor 2: Mental health	Factor 3: Substance use
1. Ever had a sexual experience	0.24		
2. Used contraceptives	0.67		
3. Used condoms	0.60		
4. Learn more about sexual health	0.71		
5. Little interest or pleasure in doing things		0.68	
6. Feeling down, depressed, irritable or hopeless		0.82	
7. Trouble falling asleep, staying asleep or sleeping too much		0.74	
8. Feeling tired or having little energy		0.75	
9. Poor appetite, weight loss or overeating		0.69	
10. Feeling bad about yourself…		0.74	
11. Trouble concentrating…		0.77	
12. Moving or speaking slowly…		0.68	
13. Thoughts that you would be better off dead		0.82	
14. Feeling nervous, anxious or on edge		0.83	
15. Not being able to stop worrying		0.32	
16. Interested in speaking to a mental health provider		0.37	
17. Used tobacco in the last year			0.70
18. Used alcohol in the last year			0.75
19. Used marijuana in the last year			0.81
20. Used vaping in the last year			0.77
21. Used prescription drugs that were not prescribed for you in the last year			0.26
22. Ever overdose			0.17

Table 4 shows the rotated component matrix returned from the EFA and summarises the correlations between each variable and its respective factor. We used varimax rotation, which keeps factors orthogonal and uncorrelated (i.e., returns unique factors that do not overlap). Each cell in the matrix represents factor loadings, with higher loadings indicating a stronger relationship between the variable and assigned factor. Variables with high loadings on the same factor are grouped, suggesting similar underlying factors. Items 1–4 loaded on a sexual health factor, Items 5–15 on a mental health factor and Items 16–22 on a substance use factor.

Factor 1 addressed sexual health, Factor 2: mental health and Factor 3: substance use. Reliability statistics were statistically acceptable for mental health and substance use (*α* = 0.91 and 0.71, respectively), but poor for sexual health (*α* = 0.41). Results from the CFA suggest a mostly good model fit based on the three‐factor solution discovered in the EFA. The CFI was 0.98, RMSEA was 0.08 and SRMR was 0.07. Cronbach's *α* for the three‐factor solution was 0.93.

### 3.2 Qualitative Results

Healthcare provider interviews explored the diagnostic accuracy of the TT Assessment. All providers (100%) reported a positive experience with the TT assessment, noting it streamlined discussions on sensitive topics like sexuality and mental health. Most providers (4 of 5) found the TT Assessment accurate. They utilised the assessment scores to guide further enquiry. One provider said the assessment was more advanced than what they had used prior and felt it allowed for elaboration.

Mental health providers noted that scores for the PHQ and GAD were accurate. They felt patients were honest and used the scores to ‘get a foothold in how we can start services’. All providers noted that the PHQ and GAD assessments were common before TT; however, they were not administered via iPad before the TT Assessment. Finally, feedback on the time required to implement the TT Assessment varied. Three providers said the assessment added to the appointment time but did not affect overall scheduling. Time added to the visit ranged from 10 to 30 minutes, depending on additional services or referrals. Respondents also noted that the TT Assessment increased efficiency and helped create awareness and support among parents, which was a positive unintended outcome.

## 4 Discussion

This study demonstrated that the TT Assessment is a statistically and clinically valid youth‐friendly health screener that facilitates the comprehensive evaluation of adolescent risk behaviours related to sexual health, mental health and substance use. The instrument addresses a gap in existing practice: the lack of comprehensive, developmentally appropriate health screening for adolescents. While the TT Assessment includes some questions from existing instruments, like PHQ‐9, it also introduces new questions to assess sexual health. Although the sexual health items did not yield acceptable Cronbach's *α* values, qualitative findings indicate that providers found the sexual health screener questions to be appropriate. The innovative nature of the instrument is also evident in how it combines relevant adolescent health topics, instead of administering three distinct tools. Lastly, automatic scoring serves as a useful method to reduce provider burden and enhance transparency during visits, so adolescents know how to interpret the information they share.

Even though some items in our analysis had weak loadings on certain factors, overall, the results suggest that the TT assessment aligns with established psychological and behavioural constructs. The results also demonstrate that the items in this analysis accurately represent sexual and mental health, as well as substance use. Thus, the TT assessment may provide a valuable framework and interventions to address issues related to sexual health, mental health and substance use among adolescents. For example, considering the high loadings on the substance use items, when incorporated into routine clinical settings, the TT assessment may be useful for identifying high‐risk behaviours and monitoring substance use trends, thereby informing treatment strategies. With the increased prevalence of substance use disorders among adolescents and young adults [32] combined with the low rates of preventive health screening in this population [33], the TT assessment offers a unique opportunity to screen for behavioural health risks that enable timely interventions or treatments, potentially informing broader public health initiatives.

Content validation indicated that both youth and healthcare providers found the questionnaire useful for extracting the health behaviour data necessary for making informed clinical decisions and deemed it a trustworthy source. Both groups preferred the electronic format due to its capacity to streamline the assessment process and offer a confidential clinical opportunity for data collection. This finding aligns with the quantitative analyses demonstrating that the TT Assessment provides clinically relevant factors in this patient population. Quality data collection is essential for disease surveillance and informing evidence‐based policies and programmes [34]. Therefore, integrating the TT assessment into electronic health records may improve data collection efforts to systematically screen for health risks, identify them early and inform targeted interventions aimed at enhancing adolescent health and well‐being.

One of the most interesting takeaways from the study was that the electronic nature of the instrument was a significant benefit, making the assessment more youth‐friendly and effective for providers. Current healthcare practice involves administering screeners electronically before a visit, typically through an adolescent's parents or guardians, verbally in clinic with a provider (when a parent/guardian is present), or on paper at intake while in the waiting room with their parent or guardian [35, 36]. By administering the TT Assessment electronically and during confidential time, adolescents have a secure method of communication that doesn't require them to disclose information to unfamiliar providers verbally. This approach aligns with the type of communication they prefer in everyday life due to social media and smartphone technology [37]. From the provider's perspective, the assessment saves time because patients can complete the screener while they wait or during vital checks. Qualitative data showed providers believed that adolescent patients answered the assessment more honestly than if the providers verbally administered the questions, which is critical for accurate diagnosis.

The TT Assessment is an innovative tool that providers can easily adopt and integrate into adolescent wellness and sick visits. The screener is one way healthcare organisations can ensure they meet the unique needs of adolescent patients. Adolescent health needs often go unnoticed until they require severe clinical intervention. The TT Assessment helps providers identify sexual health, mental health and substance use risk behaviours early, reducing the likelihood of major health challenges in adolescent patients. Additionally, providers gain a more holistic understanding of the patient and can treat and/or refer the youth to appropriate resources.

Through this study, researchers identified an additional need to screen for non‐clinical needs that provide context for the adolescent's environment. For example, questions about HPV vaccine status and dosage, as well as the current provision of mental health services, were added to the assessment after the study but did not alter the condition‐specific content of the study. Therefore, researchers determined they did not need further validation.

### 4.1 Limitations

While many validation studies consist solely of EFA and CFA, some include comprehensive psychometric testing on individual items using Item Response Theory [38]. Although Item Response Theory can uncover characteristics such as item difficulty, discrimination and functioning, we emphasised the potential for screening and diagnostic capability. We enlisted clinicians to verify whether responses to individual items and the instrument aligned with risks or diagnoses routinely identified by healthcare providers. This approach allowed us to more concretely determine if the instrument was functioning as intended: to easily identify adolescents who might require resources or access to care before a crisis or trauma. Future studies might adopt a more robust and specific approach to item validation through Item Response Theory. Another limitation of this study is the low Cronbach's *α* of 0.41 reported for the sexual health factor. While we may not fully understand why this low *α* value occurred, it is likely that the limited number of sexual health items (less than 5) may have constrained the opportunity for the items to covary strongly [39]. It is worth noting that, although the central tenets of this study—namely, sexual and mental health issues among adolescents—apply to adolescents worldwide, the tool was developed to meet the unique healthcare needs of US adolescents. Consequently, its applicability may not be feasible in international or resource‐limited settings.

## 5 Conclusion

Researchers conducted a three‐phase psychometric development and validation study to investigate the validity and reliability of the TT Assessment, an innovative tool designed to evaluate adolescent risk behaviours related to sexual health, mental health and substance use. Analysis suggested that the TT Assessment offers factors of clinical relevance in this patient population. However, there is room for further research regarding the sexual health items, as the reliability of these items in the current study is questionable. Despite the limitations of this study, the TT Assessment represents a positive step towards providing comprehensive health screening for adolescents, enabling timely preventive care and intervention.

## Author Contributions

**Whitney R. Garney:** conceptualisation, methodology, supervision, funding acquisition, project administration, writing – review and editing. **Kelly L. Wilson:** conceptualisation, methodology, project administration, writing – review and editing, supervision. **Meg Patterson:** data curation, software, validation, formal analysis, investigation, writing – original draft. **Caitlin B. Holden:** project administration, writing – original draft, writing – review and editing. **Sara Flores:** project administration, writing – original draft, writing – review and editing. **Sonya Panjwani:** project administration, writing – original draft, writing – review and editing. **Kristen Garcia:** project administration, writing – original draft, writing – review and editing. **Christi Esquivel:** project administration, writing – original draft, writing – review and editing. **Ashley Khanhkham:** project administration, writing – original draft, writing – review and editing. **Kobi V. Ajayi:** supervision, project administration, writing – original draft, writing – review and editing.

## Ethics Statement

All study recruitment and measurement procedures were approved by the Texas A&M University Institutional Review Board and by the respective clinic sites before implementation, when required.

## Consent

Active parental consent and youth assent were obtained from all participants.

## Conflicts of Interest

The authors declare no conflicts of interest.

## Data Availability

The data that support the findings of this study are available upon request from the corresponding author. The data are not publicly available due to privacy or ethical restrictions.
